# Sub-Tenon Injections of Triamcinolone Acetonide Had Limited Effect on Cystoid Macular Edema Secondary to Nanoparticle Albumin-Bound-Paclitaxel (Abraxane)

**DOI:** 10.1155/2015/181269

**Published:** 2015-08-23

**Authors:** Naoki Matsuoka, Hiruma Hasebe, Tetsuji Mayama, Takeo Fukuchi

**Affiliations:** ^1^Division of Ophthalmology and Visual Science, Niigata University, 1-757 Asahimachi-dori, Chuo-ku, Niigata, Niigata Prefecture 9518510, Japan; ^2^Murakami General Hospital, Niigata, Japan

## Abstract

*Purpose*. To report the first case of cystoid macular edema (CME) induced by nanoparticle albumin-bound- (nab-) paclitaxel treated with sub-Tenon injections of triamcinolone acetonide (STTA) with detailed long-term follow-up. *Case*. A 39-year-old Japanese woman with breast cancer presents with decreased vision in both eyes while receiving nab-paclitaxel. Two STTA treatments were administered for persistent CME in her right eye. Central retinal thickness (CRT) of the treated eye decreased after the first STTA, but there was no change after the second STTA. CRT of the other eye and bilateral visual acuity (VA) showed no change after each treatment. However, this patient experienced gradual recovery of visual function after nab-paclitaxel treatment was completed, 3 months after the second STTA. Improvements in VA and CRT did not overlap in time. Moreover, there was a big improvement time lag in VA between the eyes. *Conclusion*. Cessation of nab-paclitaxel could lead to resolution of CME more than STTA, although STTA had some effect. Since nab-paclitaxel has been recently approved for treating more types of malignancies, the number of the patients with this CME is expected to increase in the near future. Patients and physicians should understand this side effect and prepare for other treatment options.

## 1. Introduction 

Nanoparticle albumin-bound- (nab-) paclitaxel (Abraxane; Abraxis BioScience, Los Angeles, CA, USA) is used to treat various malignancies. It is associated with fewer hypersensitivity reactions than other medications in the taxane class and does not require the additional use of steroids and antihistamines. Bilateral cystoid macular edema (CME) is known to be a rare side effect of nab-paclitaxel. Several reports have described that intravitreal injections of bevacizumab [1], topical dorzolamide [2], topical steroids, and nonsteroidal anti-inflammatory drugs [3], which are used to treat typical CME, have little effect on CME induced by nab-paclitaxel. Cessation of the drug can lead to improvements in visual function [4, 5]. To the best of our knowledge, this is the first reported case of nab-paclitaxel-induced CME treated with posterior sub-Tenon injections of triamcinolone acetonide (STTA) with detailed long-term follow-up.

## 2. Case Report

**Figure 1:**
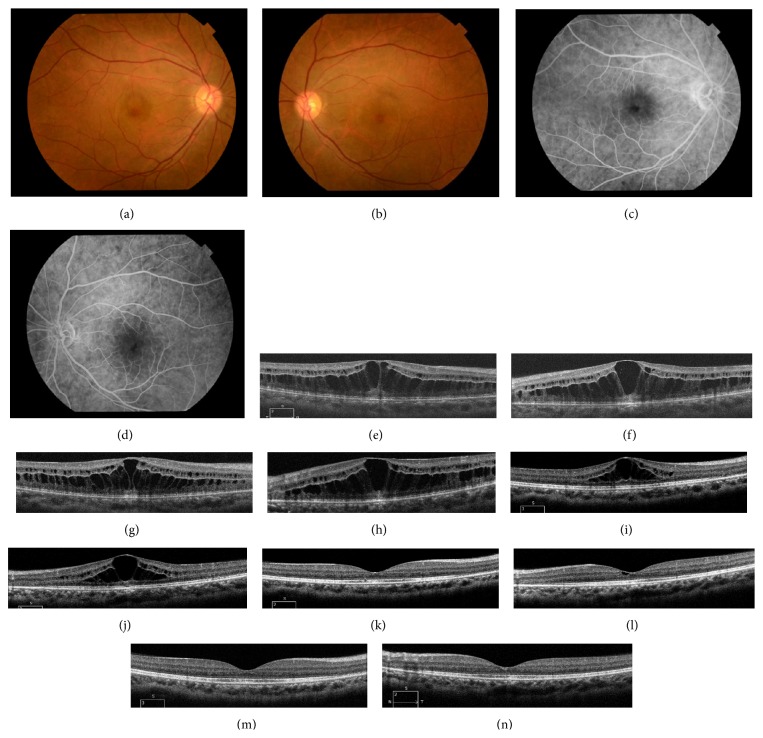
Color fundus photography showed cystoid macular edema (CME) in both eyes (a, b). Fluorescein angiography (FA) showed bilateral CME with minimal leakage during the late phase, before sub-Tenon injections of triamcinolone acetonide (STTA) (c, d). Optical coherence tomography (OCT) revealed cystic accumulation of fluid in the outer retina, particularly in the outer plexiform layer, before STTA (e, f). OCT showed slightly decreased CME in the right eye (g) and almost no change in the left eye (h) 1 month after STTA. However, the height of CME in each eye (i, j) decreased 2 months after cessation of nanoparticle albumin-bound- (nab-) paclitaxel. Complete resolution of CME in the right eye occurred 5 months after cessation of nab-paclitaxel (k), which was sustained at 6 months (m). CME in the left eye was markedly reduced at 5 months after nab-paclitaxel cessation (l) and complete resolution occurred 6 months after cessation (n).

A 39-year-old Japanese woman with breast cancer had a 4-month history of decreased vision in both eyes. She had been receiving nab-paclitaxel for approximately 12 months at a dose of 400 mg (260 mg/m^2^) every 3 weeks at the time of her initial visit to our hospital. Baseline decimal visual acuity (VA) was 0.4 in the right eye and 0.6 in the left. There were no signs of intraocular inflammation or cataracts. Fluorescein angiography (FA) showed minimal leakage in the macular area of both eyes (Figures 1(a)–1(d)) and spectral-domain optical coherence tomography (SD-OCT) revealed bilateral CME (Figures 1(e) and 1(f)). Since this patient did not take any other drugs associated with CME, she was diagnosed with nab-paclitaxel-induced CME.

**Figure 2:**
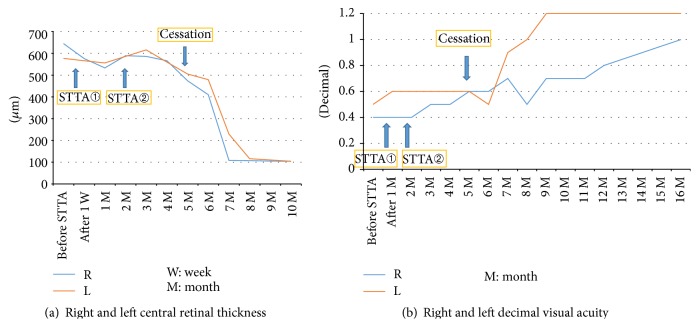
(a) Right and left central retinal thickness (CRT) over time. CRT of the right eye decreased from 645 to 533 *μ*m after the first sub-Tenon injections of triamcinolone acetonide (STTA) treatment and CRT of the untreated left eye remained relatively constant, from 577 to 556 *μ*m, during the same period. However, CRT of both eyes did not decrease after the second STTA treatment; they normalized approximately 3 months after cessation of nanoparticle albumin-bound- (nab-) paclitaxel. (b) Right and left decimal visual acuity over time. There was no change in VA of the right eye after 2 STTA treatments. It gradually recovered to 1.0 at 11 months after cessation of nab-paclitaxel. On the other hand, VA of the left eye improved more rapidly, to 1.2, at 5 months after cessation.

STTA (20 mg) was administered for persistent CME in the right eye 3 months later and a second STTA procedure (20 mg) occurred 5 months after her initial visit. Although her subjective symptoms improved slightly, there was no appreciable change in VA after the 2 STTA treatments. Her right central retinal thickness (CRT) decreased from 645 to 533 *μ*m after the first STTA treatment. In contrast, CRT of the untreated left eye remained relatively constant, from 577 to 556 *μ*m, during the same period. However, CRT in both eyes did not decrease after the second STTA treatment (Figure 2(a)).

Three months after the second STTA treatment, nab-paclitaxel treatment concluded after 23 courses because her physical status had improved. She experienced gradual recovery of her visual function after cessation of nab-paclitaxel. VA in the right eye recovered slowly to 1.0 at 11 months after nab-paclitaxel cessation. VA in the left eye improved more rapidly to 1.2 at 5 months after cessation (Figure 2(b)). SD-OCT revealed complete resolution of CME at 5 months after cessation in the right eye and at 6 months in the left (Figures 1(g)–1(n)). There were no significant differences in central sensitivity between the eyes with critical flicker-fusion frequency testing and Humphrey visual field testing at 9 and 11 months after the second STTA treatment, respectively.

## 3. Discussion

Decreased CRT in the treated right eye after the first STTA treatment may represent a minor anatomic response. However, this effectiveness was limited, as evidenced by the lack of change in VA and the reaction to the second STTA treatment. Cessation of nab-paclitaxel led to the resolution of CME more so than STTA therapy in this case.

After cessation of nab-paclitaxel, the timing of improvements in VA and CRT did not coincide. In addition, there was a big improvement time lag in VA between the eyes. The pathogenesis of CME in this patient was not clear. There is no definitive explanation of the VA and CRT results, but improvements in visual function over time and anatomic changes do not always overlap in conditions such as CME secondary to retinal vein occlusion and age-related macular degeneration. Asymmetric spontaneous resolution and the effect of STTA treatment are possible explanations of the time lag between the eyes. The right eye might have developed CME first and was more impaired at her initial visit. Nab-paclitaxel treatment for more than one year, which is longer than in previous reports [1, 3, 5], may be a potential dose-dependent risk factor for irreversible side effects.

One proposed mechanism for the pathogenesis of this type of CME is that nab-paclitaxel may be toxic to the Mueller cells, leading to intracellular fluid accumulation and subclinical leakage of extracellular fluid [6]. Some reports [5, 7] on fluid retention syndrome (FRS) [8, 9] suggest that increased capillary fluid filtration leads to capillary protein leakage and edema. However, our patient had no evidence of systemic FRS.

Paclitaxel is an antineoplastic agent in the taxane class. It targets the microtubule network in cells, which is essential for mitosis and interphase cell function [5, 10]. Paclitaxel has been shown to disrupt the microtubule structure in retinal pigment epithelium (RPE) cells by preventing microtubule disassembly, thereby blocking many microtubule-dependent processes in the RPE [11, 12]. Elevated viscosity of the exudates could be an obstacle to the diffusion of fluorescein, resulting in the absence of hyperfluorescence [11]. There are several hypothesized explanations for this type of CME with no or minimal leakage: the rate of fluid egress may be too slow to be detected by FA, or the blood-ocular barrier may be selectively impaired to molecules smaller than fluorescein [7].

Long-term cystic accumulation of fluid in the outer retina can cause irreversible visual function loss. There was no significant difference in FA, OCT findings, and results of other examinations between the eyes. The time frame to resolution of CME according to SD-OCT for this patient was similar to that of previously reported cases [1, 3, 5], which ranged from 3 to 4 months after the cessation of nab-paclitaxel.

Corticosteroids have a stabilizing effect on vascular permeability and reduce inflammatory reactions [13]. In recent years, intravitreal triamcinolone acetonide (IVTA) as an adjunct therapy for some types of macular edema has yielded promising results [14–16]. However, IVTA may provoke severe ocular complications such as endophthalmitis, retinal detachment, and secondary glaucoma [17, 18]. STTA is reported to be an alternative method that might provide a comparable effect to the IVTA with fewer complications [19, 20]. In our patient, STTA was chosen because of its stabilizing effect on vascular permeability and a safer adverse effect profile than IVTA.

In this case, CME did not resolve as we expected because the ability of TA to stabilize vascular permeability was not sufficient in the setting of no definitive evidence of intraocular inflammation.

Nab-paclitaxel was initially approved only for the treatment of metastatic breast cancer, but it has been recently approved for gastric, non-small-cell lung, and pancreatic cancer. Therefore, the number of the patients with this type of CME is expected to increase in the near future. Better understanding of the mechanisms underlying nab-paclitaxel-induced CME is needed, especially in patients with limited systemic treatment options for metastatic cancer. Patients and physicians should understand this side effect and prepare for other treatment options.
